# Application of nanotechnology in the treatment of glomerulonephritis: current status and future perspectives

**DOI:** 10.1186/s12951-023-02257-8

**Published:** 2024-01-03

**Authors:** He-Qin Zhan, Xiaoxun Zhang, Xu-Lin Chen, Liang Cheng, Xianwen Wang

**Affiliations:** 1 Department of Pathology, School of Basic Medical Sciences, Anhui Medical University, Hefei, 230032 China; 2 Department of Pathology, The Second Affiliated Hospital of Anhui Medical University, Hefei, 230601 China; 3 Department of Burns, The First Affiliated Hospital of Anhui Medical University, Hefei, 230022 People’s Republic of China; 4 Institute of Functional Nano & Soft Materials (FUNSOM), Jiangsu Key Laboratory for Carbon-Based Functional Materials and Devices, Soochow University, Suzhou, 215123 People’s Republic of China; 5 School of Biomedical Engineering, Research and Engineering Center of Biomedical Materials, Anhui Medical University, Hefei, 230032 China

**Keywords:** Nanotechnology, Nanocarrier, Primary glomerulonephritis, Diabetic nephropathy, Lupus nephritis, Therapy

## Abstract

Glomerulonephritis (GN) is the most common cause of end-stage renal failure worldwide; in most cases, it cannot be cured and can only delay the progression of the disease. At present, the main treatment methods include symptomatic therapy, immunosuppressive therapy, and renal replacement therapy. However, effective treatment of GN is hindered by issues such as steroid resistance, serious side effects, low bioavailability, and lack of precise targeting. With the widespread application of nanoparticles in medical treatment, novel methods have emerged for the treatment of kidney diseases. Targeted transportation of drugs, nucleic acids, and other substances to kidney tissues and even kidney cells through nanodrug delivery systems can reduce the systemic effects and adverse reactions of drugs and improve treatment effectiveness. The high specificity of nanoparticles enables them to bind to ion channels and block or enhance channel gating, thus improving inflammation. This review briefly introduces the characteristics of GN, describes the treatment status of GN, systematically summarizes the research achievements of nanoparticles in the treatment of primary GN, diabetic nephropathy and lupus nephritis, analyzes recent therapeutic developments, and outlines promising research directions, such as gas signaling molecule nanodrug delivery systems and ultrasmall nanoparticles. The current application of nanoparticles in GN is summarized to provide a reference for better treatment of GN in the future.

**Graphical Abstract:**

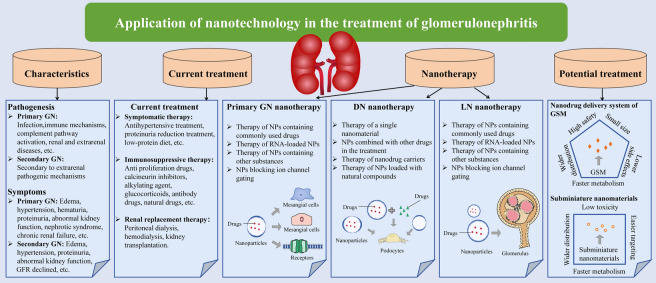

## Introduction

Glomerulonephritis (GN) is a group of inflammatory diseases characterized by glomerular injury and lesions. The causes of GN are relatively complex, and the pathogenesis is not yet clear. It may be caused by autoimmune dysfunction or may be related to multiple factors, such as genetics, infection, drugs, viruses, and the environment. GN can be divided into primary and secondary GN in terms of etiology [1]. Common pathological types of primary GN include acute GN, crescentic GN, membranous GN, membranoproliferative GN, mesangial proliferative GN, focal segmental glomerulosclerosis (FSG), minimal change GN, IgA nephropathy (IgAN) and chronic GN. The most common types of secondary GN include diabetic nephropathy (DN) and lupus nephritis (LN) **(**Table 1**)**. The symptoms of GN vary widely. Many patients have no special symptoms in the early stage. Some patients present with nephritic syndrome (hematuria, proteinuria, edema and hypertension), and some patients show nephrotic syndrome (hyperlipidemia, severe edema, heavy proteinuria and hypoproteinemia) [2]. Even patients with the same pathological characteristics may exhibit varying degrees of proteinuria [3]. However, some typical clinical manifestations can correspond to specific GN. After 1–4 weeks of streptococcal infection, patients with symptoms such as edema, hypertension, and hematuria can be diagnosed with acute GN [4]. Recurrent episodic microscopic or macroscopic hematuria is a landmark clinical feature of IgAN, which is the most common primary GN in the world [5]. Minimal changes in GN are the most common type in children [2]. Except for LN, the other types of GN are more common in the male population than in the female population [6].

**Table 1:** Disease characteristics of common glomerulonephritis

	**Disease**	**Pathogenesis**	**Symptoms**	**Treatment**
Primary GN	Acute GN	Infection, immune complex deposit circulating or implanted antigens	Abnormal urine, edema, hypertension, abnormal kidney function	Symptomatic treatment
	Crescentic GN	Immune mechanisms or renal and extrarenal diseases	Symptoms such as proteinuria and hematuria progress rapidly to oliguria and anuria	Corticosteroids, immunosuppressor, plasmapheresis
	Membranous GN	Secondary to infections, tumors, systemic immune diseases, or some drugs	Nephrotic syndrome	Spontaneous remission occurs in up to a third of patients, symptomatic treatment, immunosuppressor
	Membranoproliferative GN	Immune complex deposit or complement pathway activation	Hematuria, proteinuria, chronic renal failure or nephrotic syndrome	Symptomatic treatment, corticosteroids, immunosuppressor
	Mesangial proliferative GN	Immune complex deposit	Hematuria, proteinuria or nephrotic syndrome	Symptomatic treatment, immunosuppressor
	FSG and Minimal change GN	Undefined, may be podocyte injury or circulating permeability factor action	Nephrotic syndrome or proteinuria	Symptomatic treatment, immunosuppressor
	IgAN	Immune complex deposit or abnormal immunomodulation	Hematuria or proteinuria	Symptomatic treatment, immunosuppressor are offered to patients with a rapidly progressive course of GN
	Chronic GN	Caused by the development of acute GN or immune-mediated inflammation	Proteinuria, hematuria, hypertension, edema or chronic renal failure	Symptomatic treatment, corticosteroids, immunosuppressor
Secondary GN	DN	Oxidative Stress, hyperglycemia, inflammation and renin–angiotensin–aldosterone system	Decline in renal function, proteinuria and GFR declined	Symptomatic treatment, control blood glucose
	LN	Immune complex deposit, chemokine-mediated recruitment of different leukocyte subsets and extrarenal pathogenic mechanisms	Usually asymptomatic or proteinuria	Symptomatic treatment, corticosteroids, immunosuppressor

*GN* Glomerulonephritis, *FSG* Focal segmental glomerulosclerosis, *IgAN* IgA nephropathy, *DN* Diabetic nephropathy, *LN* Lupus nephritis

Renal biopsy is the preferred method of diagnosis for GN and can guide treatment and prognosis [7]. Many patients have no special signs in the early stage, which increases the difficulty of disease diagnosis and treatment. At the same time, increasing evidence shows that immunosuppressive drugs have limited therapeutic effects on IgAN [2]. The 1-year renal survival rate of patients who are double-positive for antineutrophil cytoplasmic antibody (ANCA) and anti-glomerular basement membrane antibody (anti-GBM) with crescentic GN, even after treatment with immunosuppressive drugs and plasma exchange, remains lower than that of patients with either anti-GBM or ANCA alone [8]. GN has a high incidence rate, which usually cannot be completely cured, and may progress to chronic GN and end-stage renal disease (ESRD) [9]. Research has shown that the incidence of ESRD may increase in the future [10]. When GN progresses to ESRD, hemodialysis or kidney transplantation is almost the last option. Dialysis therapy may cause a serious economic burden to patients [11]. In the long run, kidney transplantation may have higher economic value than dialysis, but approximately 40% of kidney transplants fail within ten years, without improving kidney and patient overall survival rates [12].

At present, the primary clinical therapy for GN is symptomatic therapy and immunosuppressive therapy (e.g. glucocorticoid, ciclosporin, etc.). Most patients with GN can only delay the progression of the disease and cannot be completely cured. Some types of patients with GN are prone to relapse and steroid resistance after treatment, or progress to ESRD, which are very difficult to treat. Patients with ESRD are usually treated with replacement therapy, which increases the economic burden of patients and reduces the quality of life. The side effects of some immunosuppressants exceed the therapeutic effect. Therefore, new GN treatment methods are urgently needed [1, 7].

**Fig. 1:**
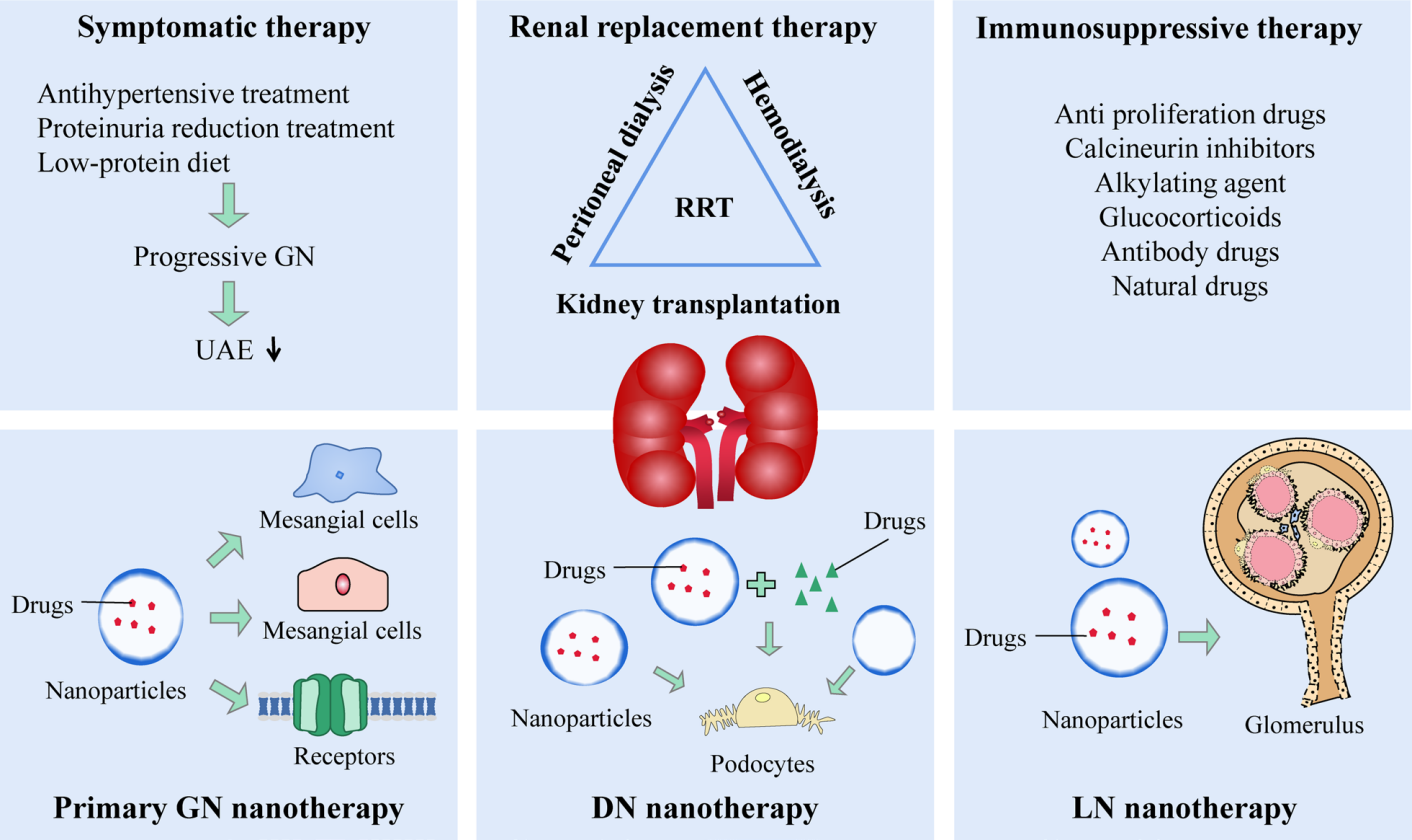
Schematic diagram of GN treatment. (Symptomatic therapy, renal replacement therapy, immunosuppressive therapy, primary GN nanotherapy, DN nanotherapy, LN nanotherapy). *GN*  glomerulonephritis; *DN* diabetic nephropathy; *LN* lupus nephritis; *RRT* renal replacement therapy; *UAE* urinary albumin excretionopathy

Nanotechnology has flourished in multiple fields, including medicine, such as targeted transportation, in vivo imaging, assisted diagnosis, and disease treatment. Encapsulating drugs with nanoparticles (NPs) can target drug delivery, improve bioavailability, avoid systemic effects, and reduce drug toxicity and adverse reactions [13]. Specific dimensions can be more effectively deposited on the target area and play a targeted role. There have been many attempts to deliver drugs to the kidney by nanodrug loading systems, which mainly deliver drugs to mesangial cells, podocytes and endothelial cells [14]. Although some reviews have summarized the application of nanodrugs in kidney diseases or DN [15, 16], there is no review related to nanodrugs in the treatment of GN. Therefore, this review briefly introduces the concept, pathologic changes and current treatment situation of GN, summarizes the application of nanotechnology in the treatment of primary GN and secondary GN (DN and LN) **(**Fig. 1**)**, and proposes some treatment methods that have the potential to be applied in the treatment of GN. The focus of this review is on the application and prospects of nanotechnology in the treatment of GN.

## Changes in the glomerular filtration barrier (GFB) in GN

GFB consists of glomerular endothelial cells, glomerular basement membrane (GBM) and podocytes. Podocytes originate from the renal mesenchyme and reach the outside of the GBM. Structural changes in podocytes may cause kidney diseases, including GN. For example, patients with DN lose and damage podocytes, resulting in GFB damage and kidney lesions.

### Physiological and pathophysiological structure of GFB

GFB is a unique structure with selective permeability, serving as both a molecular barrier and a charge barrier, which can prevent the filtration of various plasma albumin and negatively charged macromolecules in the blood. Proteinuria, which occurs when the GFB is damaged, is characteristic of most kidney diseases and is a factor in the rapid progression to renal failure [17].

### Glomerular hyperfiltration

**Fig. 2:**
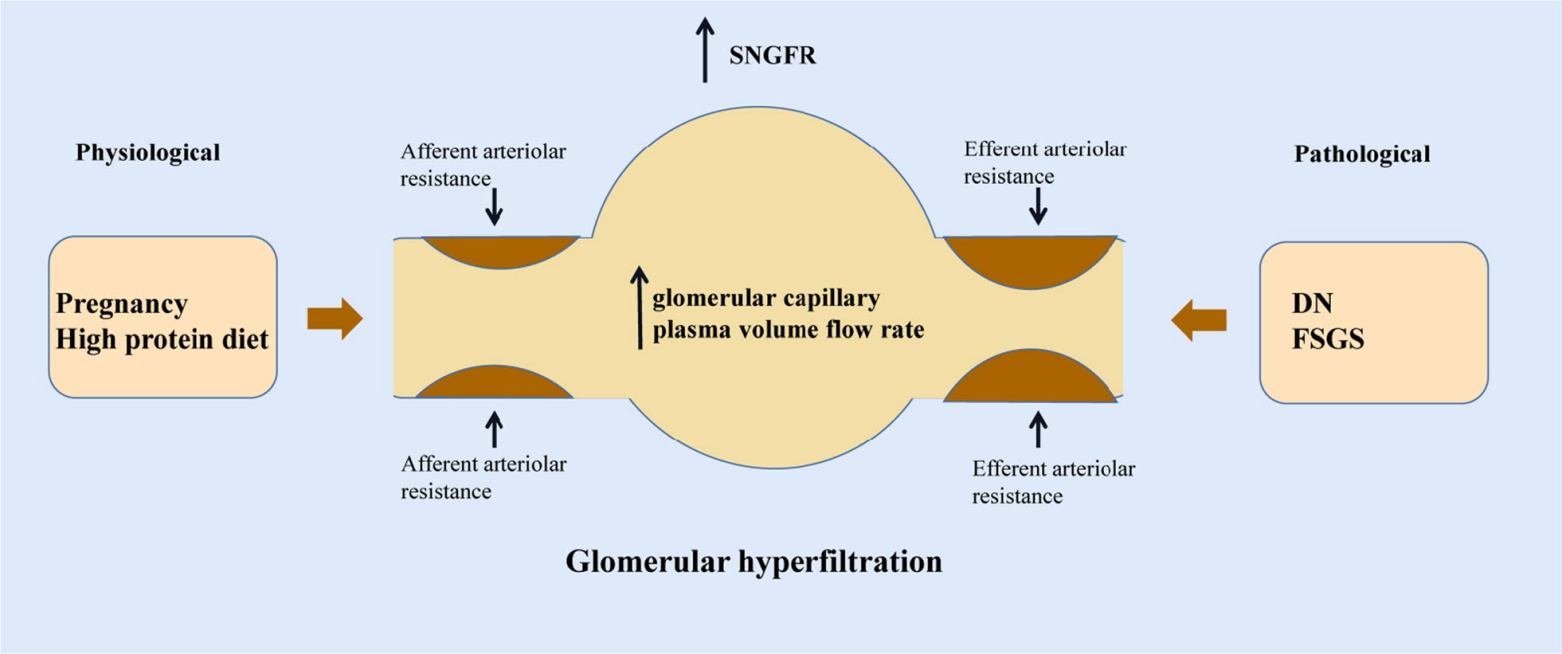
Factors in glomerular hyperfiltration. The factors of glomerular hyperfiltration include physiological glomerular hyperfiltration and pathological glomerular hyperfiltration, among which physiological factors include a high-protein diet and pregnancy, and pathological factors include DN and FSGS. *SNGFR* single-nephron glomerular filtration rate; *DN* diabetic nephropathy; *FSGS* focal segmental glomerulosclerosis

Glomerular hyperfiltration is an abnormal increase in the glomerular filtration rate (GFR), filtration fraction or nephron filtration. A high-protein diet and pregnancy can cause physiological glomerular hyperfiltration [18, 19]. In DN, changes in renal function successively lead to glomerular hyperfiltration, increased urinary protein and decreased progressive GFR. Glomerular hyperfiltration may be an early manifestation of DN. Hyperglycemia may be a factor in the pathogenesis of glomerular hyperfiltration in patients with diabetes, and changes in renal function related to blood glucose have important significance for the progression of DN. At the same time, glomerular hyperfiltration was also observed in the secondary FSG (Fig. 2).

In summary, GFB plays a crucial role in maintaining renal homeostasis. An abnormal increase in GFR and destruction of the GFB are important factors affecting the development and poor prognosis of GN. Repairing and protecting the structure of the GFB and reducing glomerular hyperfiltration are therapeutic strategies for GN.

## Current treatment status of GN

GN is the main cause of chronic kidney disease. Therefore, GN patients need symptomatic treatment to delay disease progression. The disease classification of GN lacks complete consensus in histopathology. Different classifications of GN based on pathological or etiological definitions have the same clinical manifestations, and the treatment method is determined based on clinical manifestations [1].

### Symptomatic therapy

Symptomatic treatment is applicable to all progressive GN, including antihypertensive treatment, proteinuria reduction treatment and a low-protein diet. ACE inhibitors (ACEis) or angiotensin II receptor blockers (ARBs) are first-line drugs for treating hypertension and proteinuria [7, 20]. Symptomatic treatment can alleviate the symptoms of proteinuria and slow the progression of nonspecific GN. In adult GN patients with nephrotic syndrome, antiedema and anticoagulant therapy also need to be considered. Based on the evaluation of patient status, serum albumin levels below 20–25 g/L may indicate anticoagulation prevention [21]. For edema symptoms, loop diuretics are the first choice, and a low salt diet is needed [7]. For IgAN, because of the typical clinical manifestations, such as massive proteinuria, hypertension and reduced GFR, oral glucocorticoids combined with ACEis or ARBs are the first-line treatment [22]. Immunosuppressive drugs should not be used except for those with rapidly progressing IgAN [2].

### Immunosuppressive therapy

In addition to symptomatic treatment applicable to all patients, immunosuppressive treatment methods, including glucocorticoids, calcineurin inhibitors (ciclosporin and tacrolimus), anti-proliferation and anti-metabolism drugs (mycophenolate ester), antibody drugs (baliximab and rituximab), alkylating agents (cyclophosphamide) and natural drugs (tripterygium wilfordii and its derivatives), are applicable to specific GN cases.

Glucocorticoids are first-line drugs for GN that can be applied in almost all types of GN and can effectively reduce urinary protein levels. Some studies have shown that glucocorticoids are beneficial for GN patients with proteinuria exceeding 3 g/day and a GFR below 50 mL/min [23]. Glucocorticoids are often used for minimal change GN and FSG, and FSG requires long-term high-dose treatment [1, 7]. The treatment of proliferative LN (class III and IV) and anti-GBM crescentic GN mostly starts with steroid impulse therapy, followed by oral cyclophosphamide and prednisone [1].

In membranous GN, alkylating agents (such as cyclophosphamide) plus corticosteroids or calcineurin inhibitors are commonly used for treatment. Immunosuppressive drugs are only considered when GFR decreases or severe complications of nephrotic syndrome occur [2]. Rituximab is a relatively new treatment method that has achieved partial or complete remission in membranous GN [24] and may have a direct impact on podocytes, thus having a certain therapeutic effect in minimal change GN [25]. Triptolide can inhibit the proliferation of mesangial cells in IgAN, and its application in membranous GN, LN and DN can reduce proteinuria and play a role in renal protection [26–30].

### Renal replacement therapy

Although there are currently treatment and remission programs for GN and researchers are constantly making new attempts, there are still many GN patients whose conditions cannot be controlled and gradually progress to chronic GN and even end-stage renal failure. For these patients, renal replacement therapy is a method of life support and life assurance treatment. Renal replacement therapy includes hemodialysis, peritoneal dialysis and kidney transplantation [31]. Patients use dialysis to remove excess body fluids to restore normal circulation and blood pressure. Different dialysis membranes are used according to the actual situation to remove impurities and fluids. Most patients undergo dialysis three times a week. Patients can maintain a normal life through dialysis treatment, but not all patients can achieve good results. Infection, malnutrition and other factors can all cause complications of hemodialysis, with infection being one of the main causes of death in hemodialysis patients [32].

Renal transplantation is the last resort for end-stage renal failure. It can improve the survival rate and quality of life of patients with renal failure. After transplantation, immunosuppressive drugs are required to maintain the status of the transplanted kidney [33]. Although kidney transplantation can be successful, transplant patients are prone to recurrent GN, thus leading to transplant failure [34].

## Application of nanomaterials in GN treatment

Nanomaterials are widely used in kidney diseases and can be used for disease diagnosis, detection of renal structure and function, delivery of drugs, and prevention of diseases (such as transplant-induced reactions) [13]. Researchers are committed to developing NPs with both diagnostic and therapeutic functions [35]. NPs are effective carriers that can carry various types of substances and can be targeted for delivery to kidney tissue and cells, achieving better results than conventional therapy and reducing the systemic effects of drugs and adverse reactions [36].

### The role of nanomaterials in the treatment of primary GN

**Fig. 3:**
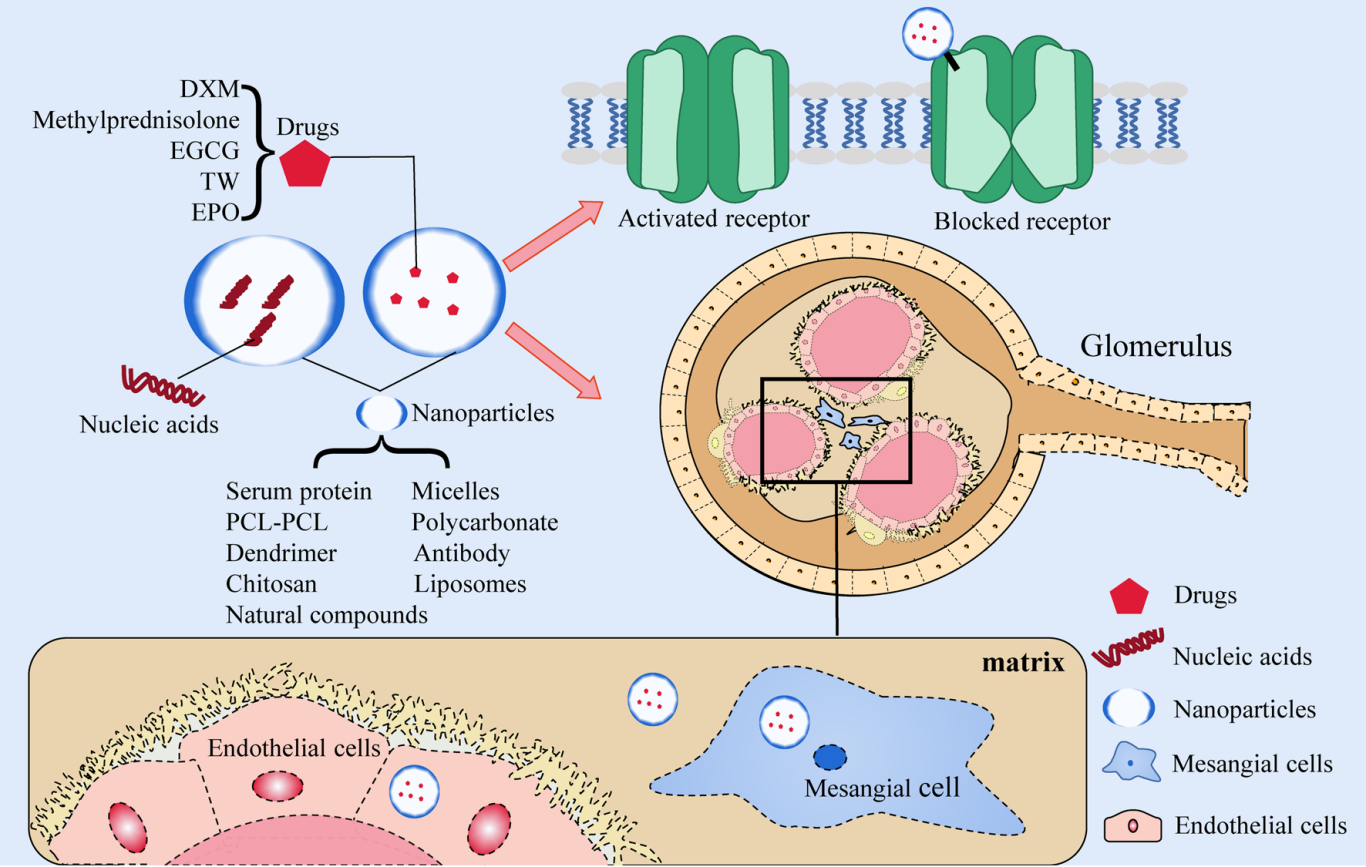
Treatment of primary GN with nanomaterials. In the treatment of primary GN, a nanodrug delivery system can deliver various drugs and nucleic acids to mesangial cells, endothelial cells and the mesangium. Nanoparticles can also play a therapeutic role by blocking ion channels. *GN* glomerulonephritis, *PCL-PCL* polyethylene, glycol-polycaprolactone, *DXM* dexamethasone, *EGCG* epigallocatechi gallate, *TW* tripterygium wilfordii; *EPO* erythropoietin

In the treatment of primary GN, NPs typically function as a delivery system capable of transporting various drugs and nucleic acids. Additionally, they can modulate ion channel gating to regulate inflammation (Table 2) (Fig. 3).

**Table 2:** Application and efficacy of nanomaterials in primary glomerulonephritis

Disease	Material	Particles	Target	Therapeutic Effects	Ref
GN	Human serum protein peptide fragments	Peptide fragment of Triptolide (immunosuppressive)—human serum albumin:PF-A1-123,PF-A124-298 and PF-A299-585	Kidney	Reduce cytotoxicity, relieve symptoms of membranous nephropathic	[41]
MSPGN	Albumin	CLT-loaded albumin nanoparticles	MC	Alleviate proteinuria, inflammation, glomerular hypercellularity, and excessive extracellular matrix deposition	[40]
MN	Liposomes	3,5-dipentadecyloxybenzamidine hydrochloride (TRX-20)-modified liposomes	MC	Relieve symptoms of membranous nephropathic, improved proteinuria, serum cholesterol and albumin	[42]
GN	Lipid	Lipid based nanocarrier system (Saint-O-Somes)	Podocytes	Targeted drug delivery to podocytes	[43]
GN	Micelles	Novel Biomimetic pH Sensitive Nanomicelles Loaded with DXM(MM/HA-DXM)	MC	Improve targeting performance, reduce proteinuria, have anti-inflammatory effect	[46]
MsPGN	Polyethylene glycol-polycarbonate	Polyethylene glycol-polycarbonate nanoparticles loaded with DXM	MC	Drugs can accumulate more in the kidney and renal cortex	[47]
MGN	Liposomes	Methylprednisolone bovine serum albumin nanoparticles (ME BSA NPs)	Mesangium	Reduce the levels of 24 h urinary protein and serum creatinine	[48]
GN	Polycarbonate	DXMS and CAP loaded PLGA-IL delivery system (called DXMS/CAP@PLGA-ILs)	MC	Improve the pathological changes in the mesangial area and positive expression of proliferating cell nuclear antigen in glomeruli	[52]
GN	L-serine (Ser)-modified polyamidoamine dendrimer (PAMAM)	L-serine modified polyamide dendronized polymer loaded with CAP	Renal cortex	Targeted drug delivery	[53]
GN	Polycationic cyclodextrin	Polycationic cyclodextrin nanoparticles containing siRNA (siRNA/CDP-NPs)	Mesangium	Targeted drug delivery to mesangial cells	[58]
MsPGN	Chitosan	Chitosan nanoparticles containing xPDGF-Band PDGFR-β	MC	Reduce mesangial cell proliferation and matrix accumulation	[59, 60]
IgAN	Liposomes	Size and Surface charge dependent glomerular targeting liposome nanoparticles containing p38e and Surface csiRNA	MC and endothelial cell	Alleviate proteinuria, inflammation and excessive extracellular matrix deposition	[61]
GN	Cationic liposome	Cationic liposomes loaded with PAI-1R	Glomeruli	Reduce increases in glomerular matrix accumulation and expression of PAI-1R and fibronectin	[62]
GN	Au/Liposomes	Gold nanoliposome loaded with DXMS and TGF wiAu-ILs)	Mesangium	Inhibit local inflammation and fibrosis, produce better therapeutic effects	[63]
GN	Natural compounds	Fucoidan nanoparticles	Renal cells	Improve the necrosis of renal cells, decreases BUN, creatinine, MDA, IL-6, and TNF-Num > < Disp	[64]
NS	Natural compounds	PH sensitive EGCG nanoparticles	Renal cells	Reduce proteinuria and improve renal pathological changes	[65]
GN	Antibody	Nanoantibodies of P2X7	Ion channel	Improve drugs release efficiency and relieve experimental glomerulonephritis	[70]

*GN* glomerulonephritis, *MSPGN* mesangial proliferduringive glomerulonephritis, *MC* Mesangial cells, *MsPGN* mesangial proliferative glomerulonephritis, *MGN* mesangial glomerulonephritis, *IgAN* IgA nephropathy, *NS* nephrotic syndrome, *MN* membranous nephropathy, *DXM* Dexamethasone, *NPs* nanoparticles, *CAP* captopril, *PAI* plasminogen activator inhibitor

#### The therapeutic effect of NPs containing commonly used drugs on primary GN

In China, tripterygium wilfordii (TW) and its formulations have been used for the treatment of GN [37]. Human serum albumin (HSA), as the most abundant protein in plasma, has many excellent properties and is widely used in biomedical fields [38]. By cleaving HSA into small molecules that can be filtered by glomeruli, peptide fragments (PFs) of HSA can accumulate in the kidney and be reabsorbed by proximal tubular cells after glomerular filtration. Such PFs can be suitable kidney-targeting carriers [39]. Celastrol (CLT) is an immunosuppressive and anti-inflammatory agent derived from TW. CLT-loaded albumin NPs with a size of 95 nm can target drug delivery to mesangial cells and then achieve maximum accumulation, thus reducing CLT-related systemic toxicity and alleviating the symptoms of proteinuria. It also decreases the mRNA levels of MCP-1 and ICAM-1 to block monocyte infiltration and reduces the levels of IL-6 and IL-1β, thus achieving anti-inflammatory effects [40]. Triptolide (TP), which is also a bioactive diterpenoid epoxide derived from TW, has immunosuppressive and anti-inflammatory functions. TP coupled with PF-A_299-585_ (PF with amino acid sequence A_299-585_) can be targeted to the kidney for therapeutic effects, and its cytotoxicity is significantly lower than that of the free drug [41]. Similarly, 3,5-dipentadecyloxybenzamidine hydrochloride-modified liposomes specifically target mesangial cells in the glomerulus. When TP was sealed into liposomes to form drug-loaded nanoliposomes for therapeutic use, it was shown that the nanoliposomes could reverse inflammation in rodent models of nephropathy [42]. Long-term use of sirolimus as a commonly used immunosuppressive drug may lead to severe proteinuria. The lipid-based nanocarrier system can deliver sirolimus to podocytes in a targeted manner, reduce the dosage of application, and thus reduce the risk of serious side effects [43].

Glucocorticoids, which have anti-inflammatory effects, are currently the most commonly used drugs for the treatment of GN. However, the application of glucocorticoids can cause adverse effects on many organ systems, such as osteoporosis, femoral necrosis, hyperglycemia, hypertension, increased risk of infection, Cushing’s syndrome, and gastrointestinal bleeding [44]. Compared with traditional drug delivery modes, nanomaterials as transport carriers for targeted therapy have great superiority. For example, polymeric nanomicelles have the advantages of high structural stability, massive loading of hydrophobic drugs, more effective targeted transport ability and stimulation sensitivity [45]. In one study, pH-sensitive nanomicelles loaded with drugs were evaluated for the treatment of GN. By attaching dexamethasone (DXM) to hyaluronic acid (HA) and then covering the surface with natural macrophage membranes (MM), biomimetic nanomicelles (MM/HA-DXM) were formed. For the active homing action of macrophages to the inflammation site, MM/HA-DXM can be targeted for transport to the inflammation site of glomeruli. In the weakly acidic GN microenvironment, HA and DXM can be targeted for release. DXM has an inhibitory effect on mesangial cell proliferation, HA can remodel the phenotype of macrophages from the proinflammatory M1 phenotype to the anti-inflammatory M2 phenotype, and both have synergistic anti-inflammatory effects. As demonstrated by in vivo experiments, MM/HA-DXM treatment significantly increased albumin and TP levels in the treated group, while blood urea nitrogen (BUN), creatinine, cholesterol and triglyceride levels were significantly reduced, and proteinuria levels decreased up to 2.33-fold compared to the control group [46].

In another study, Li et al. [47] designed glomerulus mesangium-targeted poly(ethylene glycol)-poly(D,L-lactic-co-glycolic acid) NPs loaded with DXM acetate for the treatment of mesangial proliferative GN. The drug delivery system was able to effectively target the drug into the glomerular mesangium membrane, and NPs with a size of 90 nm had maximum accumulation in the renal mesangium membrane. Similarly, the efficacy of methylprednisolone bovine serum albumin NPs in the treatment of mesangial proliferative GN was evaluated. After nanoparticle treatment, rats with cationic bovine serum albumin-induced mesangial proliferative GN showed a significant reduction in 24-h urinary protein volume and serum creatinine, demonstrating the therapeutic effect of this drug system on mesangial proliferative GN [48].

Chronic GN is the end stage of the development of different types of GN, and renal fibrosis inevitably occurs [49]. The use of glucocorticoids does not have an antifibrotic effect, which is detrimental to the prognosis of GN. Therefore, it makes sense to simultaneously perform anti-inflammatory and antifibrotic modulation. ACEis/ARBs are also commonly used in renal diseases due to their anti-inflammatory [50] and antifibrotic functions [51]. Zhou et al. [52] constructed a DXM and captopril (CAP)-loaded PLGA-IL delivery system. The results of in vivo anti-inflammatory/anti-fibrotic treatment showed that all types of cell infiltration and proliferation were effectively controlled in PAS-stained sections in the experimental group, and the expression levels of TNF-α, IL-6, IL-β1, TGF-β, α-SMA and Fn were reduced. In addition, an l-Ser-modified polyamidoamine dendrimer as a targeted drug carrier could successfully transport CAP to the kidney [53]. Hydrophobically modified glycol chitosan (HGC) nanomicelle-delivered olmesartan to the kidney attenuated renal fibrosis in Col4a3-/- mice and did not alter their blood pressure [54].

#### Therapeutic effect of RNA-loaded NPs on primary GN

RNA interference is a process that can efficiently and specifically silence the expression of almost any gene, including endogenous microRNAs (miRNAs) and synthetic short interfering RNAs (siRNAs). By mediating targeted mRNA degradation or mRNA translation inhibition, the expression of target genes can be knocked down in a sequence-specific manner. siRNA is more advantageous than miRNA in triggering specific gene silencing [55, 56]. siRNA molecules must be delivered to the interior of the target cell to activate the RNA interference pathway, so vectors or chemical modifications are required to enter the interior of the target cell [57].

Zuckerman et al. [58] verified that intravenous injection of polycationic cyclodextrin NPs containing siRNA rapidly accumulated in the glomerular basement membrane. Several studies have shown that chitosan acts as a siRNA delivery system to target siRNA molecules of PDGF-B and PDGFR-β to the kidney. The results showed that silencing the PDGF-B signaling pathway could inhibit the proliferation of mesangial cells and has promise for the treatment of mesangial proliferative GN [59, 60]. In addition, liposome NPs loaded with p38α MAPK and p65 siRNA successfully alleviated proteinuria, inflammation and excessive extracellular matrix deposition in a mouse IgAN model [61]. Targeted application of this nanoliposome in a rat model of nephritis inhibited glomerular TGF-β gene expression, thereby specifically suppressing glomerulosclerosis [62]. In another study, similarly, NPs loaded with DXM and TGFβ1 siRNA effectively reduced the expression levels of cytokines such as TNF-α and TGF-β1, mediating the dual regulation of inflammation and fibrosis [63].

#### Therapeutic effect of NPs containing other substances on primary GN

Natural compounds often have poor oral availability issues. Nanomodels that can selectively release or target drugs are an effective way to improve drug utilization. Many studies are exploring the treatment of GN, such as administering fucoidan NPs at a dose of 300 mg/kg body weight, which can significantly reduce urea, creatinine, MDA, IL-6, and TNF-α levels, thereby improving the necrosis of renal cells [64]. In a rat model of nephrotic syndrome, epigallocatechin-3-gallate (EGCG) NPs reduced the amount of urine protein. The bioavailability of EGCG NPs was more than 2.4 times higher than that of the EGCG powder group [65].

Erythropoietin (EPO), which is produced by capillary-lined cells around the renal tubules, can modulate the production of red blood cells and is considered a drug that can treat kidney diseases [66]. Zhang et al. [67] established a rat model of IgAN and verified the therapeutic effect of EPO-loaded chitosan-triphosphate NPs for IgAN, with a significant reduction in urea and creatinine levels.

#### NPs blocking ion channel gating

Ion channels are ideal therapeutic targets, but highly specific targeted drugs are needed. P2X7 is a trimer ion channel gated by extracellular ATP that plays a role in the release of inflammatory molecules, cell proliferation and death, metabolic events and phagocytosis [68]. The activated P2X7 receptor can open pores and allow the passage of organic ions [69]. In one study, the anti-inflammatory effect of nanoantibodies targeting mouse P2X7 was evaluated. The results showed that injection of these nanoantibodies into mice could block (nanoantibody 13A7) or enhance (nanoantibody 14D5) gating of channels, prevent inflammation caused by ATP binding to P2X7 released by injured and dying cells, and improve the clinical parameters of GN. At the same time, the nanoantibody Dano1 was 7 times more effective in inhibiting the release of cellular inflammatory messengers in the human body than small molecule P1X2 antagonists [70].

### Application of nanomaterials in the treatment of DN

**Fig. 4:**
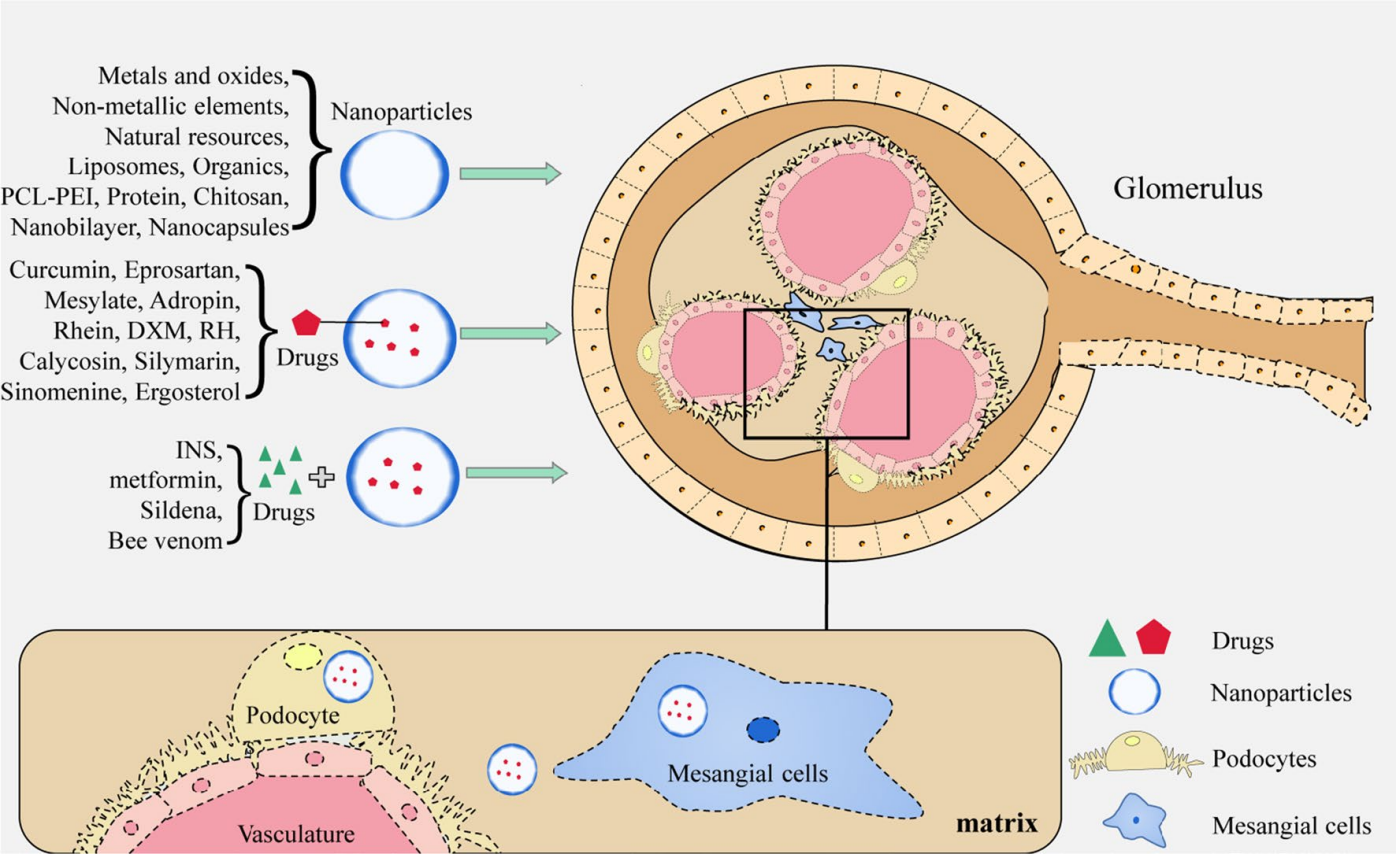
Treatment of DN with nanomaterials. In the treatment of DN, the therapeutic forms of nanoparticles include the therapeutic effect of nanoparticles themselves, the combination of nanoparticles and drugs, and the nanodrug delivery system, which can target podocytes, mesangial cells and mesangium. *DN* diabetic nephropathy, *PCL-PEI*  polycaprolactone-polyethyleneimine, *RH*  rhein; *DXM*  dexamethasone

In DN, hyperglycemia and oxidative stress are two related processes and the focus of research, and many studies are based on these two points. The forms in which NPs play a role in DN include the therapeutic effects of nanomaterials themselves, the combination of nanomaterials with drugs, and nanodrug delivery systems (Table 3) (Fig. 4).

**Table 3:** Application and efficacy of nanomaterials in diabetes nephropathy

Material	Particles	Loaded substance	Target	Therapeutic Effects	Ref
Se	SeNPs		Kidney	Reduce rate of urination, urea, creatinine, MDA and glucose	[77]
Zn	ZnONPs		Podocyte	Improve renal functionality; inhibit renal fibrosis, oxidative stress, inflammation and abnormal angiogenesis, and delay the development of podocyte injury	[81]
Zn	ZnONPs		Kidney	Improve uric acid, creatinine, BUN and urinary albumin	[82]
Ag	AgNPs			Anti-inflammatory action, reduce the serum levels of TNF- 、IFN-e、IL-17A、IL-6 and MCP-1	[85]
Au	AuNPs		GBM and podocyte	Attenuate hyperglycemia, reduce 24-h urinary albumin excretion rate, glomerular basement membrane thickness, foot process width and renal oxidative stress markers	[87]
Metal organic frameworks	Metal organic framework containing chromium			Reduce HOMA-IR index, blood urea nitrogen, uric acid and malondialdehyde in plasma samples	[89]
Metal	Nanochelating nanomedicine with iron chelating properties(BCc1)		GBM, podocyte, and tubules	Reduce albumin, malondialdehyde and 8-isoprostane in urine specimen, increased creatinine clearance	[91]
Natural resources	Nanoparticle Curcumin (nCUR) combined with INS	Curcumin/Combined INS	Kidney	Reverse or delay the histological changes of renal injury and delay progress of DN	[93]
Chitosan/Se	Metformin and chitosanselenium anoarticles (Ch SeNPs)	Combined metformin	Kidney	Inhibit oxidative stress and restore glucose hemostasis	[94]
Se	Combination of selenium nanoparticles and Sildenafil	Combined Sildenafil	Kidney	Improve renal function and histopathological changes, have protective effect	[95]
Se	Bee venom combined with SeNPs	Combined Bee venom	Kidney	Protective role against the long-term diabetic complications of DN	[96]
Se	Rutin combined with SeNPs	Combined	Kidney	Reduce fasting blood glucose, serum creatinine and urea, renoprotective effect against DN	[97]
HMSN	HMSN loaded with Metformin	Etformin	Kidney	Increase that accumulation of drugs in the kidney, relieve DN symptoms	[98]
Nanobilayer	Nanobilayer loaded with eprosartan eesylate	Eprosartan Mesylate		Decrease in serum creatinine, urea, lactate dehydrogenase, total albumin and malondialdehyde	[100]
Nanocapsules	reactive oxygen species (ROS) responsive nanocapsules (Ad@Gel)	Adropin	Glomerular endothelium	Control blood glucose and lipid levels, improve renal function, inhibit excessive production of ROS, protect mitochondria from damage, improve lipid deposition in renal tissues	[101]
PCL-PEI	Polycaprolactone Polyethylenimine (PCL-PEI) core and kidney targeting peptide (KTP) modified lipid layer	Rhein	MC and glomeruli	Reduce BUN, creatinine, fibronectin and collagen, elevate albumin	[75]
Engineering polymer	Engineering Polymer Nanoparticles Loaded with DXM	DXM	Podocyte	Repair damaged podocytes	[103]
Composite nanomaterials	MicroRNA-Gated polymer Nanocomposite	MicroRNA	Podocyte	Targeted delivery of exogenous miRNA to podocytes	[104]
Chitosan	ATRA chitosan/Triphosphoric acid lipid hybrid nanoparticles	ATRA	Kidney	Reduce of creatinine, urea, TNF- lipid hybrid nan and VEGF, elevate AMPK and LKB1	[105]
Protein	KT Targeted sHDL/TO Nanodisk(KT-sHDL/TO)	Liver X receptor agonist	MC	Suppress mesangial cell proliferation, ameliorate dyslipidemia and inflammation	[106]
PCL-PEI/natural resources	Polymer loaded with RH γ- Glutamic acid coated Polycaprolactone Polyethylenimine nanoparticles (RGPP)	RH	MC and glomeruli	Drugs accumulation in kidney	[109]
PCL-PEI	Renal targeted RH lipid nanoparticles with egg yolk shell structure(KLPPR)	RH	Kidney	Reduce the parameters of urea nitrogen, serum creatinine and kidney index, improve the urinary creatinine and the creatinine clearance rate	[110]
PEG/natural resources	RH loaded polyethylene glycol co caprolactone co Ethanimine nanoparticles(PPP-RH-NPs)	RH	MC and glomeruli	Targeted drug delivery, decrease the levels of FBG, creatinine, BUN, urine protein and the intensity of oxidative stress	[112]
PEG/natural resources	Ultra small polymer nanocarriers for drug delivery	DXM	Podocyte	Repair damaged podocytes	[113]
Organics	Deoxycholic acid conjugated nanoparticles (DNPs)	RH	Apical sodium dependent Bile acid transporters in the small intestine	Enhance oral bioavailability	[114]
Natural resources/Liposomes	Calycosin loaded nanoliposomes	Calycosin	Kidney cell mitochondria	Restore function of mitochondria, improve diabetic nephropathy	[115]
Natural resources/Liposomes	Silymarin loaded nanoliposomes	Silymarin	Podocyte	Reduce body weight/kidney ratio, renal functions and lipid profiles in renal tissues	[117]
Natural resources/Liposomes	Sinomenine loaded nanoliposomes	Sinomenine	Kidney	Improve renal function, and have renal protective effect	[116]
Natural resources/Liposomes	Nanostructured lipid carriers loaded with ergosterol	Ergosterol	Kidney	Improve oral bioavailability and therapeutic efficacy	[118]

*NPs* nanoparticles, *RH* rhein, *GBM* glomerular basement membran, *INS* long-acting insulin, *HMSN* Hollowmsoporous nano composite, *DXM* Dexamethasone, *PEG* polyethylene glycol, *PCL-PEI* Polycaprolactone-polyethyleneimine, *ATRA* all-trans retinoic acid

#### Therapeutic effect of a single nanomaterial on DN

Many studies are based on hyperglycemia and oxidative stress in DN. Selenium (Se) is an essential trace element for maintaining the basic physiological health of the human body, with functions such as redox regulation, immune response regulation, and control of inflammatory reactions [71, 72]. At the same time, the dosage and form of Se, as well as the lack and excessive intake, can have an impact on human health [73, 74]. Compared with Se and its compounds, SeNPs have smaller volumes, higher bioavailability, lower toxicity, and targeting properties [75, 76]. The results of a previous study showed that SeNPs effectively reduced the levels of BUN, creatinine, albumin, fibronectin, and collagen in DN. DN can be prevented by inhibiting oxidative stress and activating the cell protective protein HSP70 and longevity protein SIRT1 [75]. Similarly, SeNPs can play a renoprotective role in streptozotocin (STZ)-induced diabetes rodent models during pregnancy [77].

ZnONPs are the most common metal oxide nanomaterials, with unique advantages such as improving bioavailability and crossing biological barriers [78, 79]. At present, the medical field has studied the anti-inflammatory, antioxidant stress, antibacterial, and antitumor effects of ZnONPs [80, 81]. In some studies exploring the treatment of STZ-induced DN in rat models with ZnONPs, it has been demonstrated that ZnONPs can improve renal function by reducing the profibrotic cytokine TGF-β1, increasing the expression of MMP-9 and preventing renal fibrosis [81]. ZnONPs can also prevent inflammation by weakening the activation of the NLRP3 inflammasome, inhibiting TXNIP gene expression, and upregulating Nrf2 to suppress oxidative stress [82].

Some studies have shown that precious metal NPs such as AuNPs and AgNPs have anti-inflammatory and antioxidant activities [83, 84]. Among them, AgNPs have unique antibacterial, anticancer, and antiangiogenic properties and are widely used in multiple fields [84]. When inflammation was induced in pregnant mice exposed to AgNPs with STZ, their fetuses exhibited the ability to resist inflammation and kidney damage that persisted until adulthood. After DN was induced in these offspring by STZ, histopathological analysis showed that the damage to renal tubules and glomeruli was significantly reduced compared with that in the control group. This demonstrated the renoprotective effect of AgNPs [85]. AuNPs have been proven to have hypoglycemic effects in STZ-induced diabetes animal models [86]. Alomari et al. [87] confirmed that the use of AuNPs alone could improve podocyte damage and significantly reduce blood sugar and urine protein levels.

Metal organic frameworks have been widely used in various fields, including in the medical field [88]. A study demonstrated that metal organic frameworks effectively reduced the HOMA-IR index, BUN, uric acid and malondialdehyde in plasma samples of experimental DN [89]. At present, research has proven that BCc1, a nanochelate with iron chelating properties, has antitumor effects [90]. Fakharzadeh et al. [91] studied the therapeutic effect in an animal model of DN. The results indicated that the material effectively reduced urinary protein and the albumin/creatinine ratio and alleviated pathological changes in the glomerulus. The above findings suggest that NPs with anti-inflammatory and antioxidant stress properties can improve the renal injury caused by DN and have the potential for renal protection.

#### NPs combined with other drugs in the treatment of DN

Reducing blood sugar and blood pressure is an important link in the treatment of DN. However, there is also evidence that anti-inflammatory treatment has an effect on DN. Curcumin, as a natural compound, has been proven to have anti-inflammatory effects. Curcumin and its preparations can delay the progression of DN [92]. However, curcumin has low oral availability and is widely metabolized throughout the body, which makes it difficult to completely treat DN. Ganugula et al. [93] used nanocurcumin in combination with long-acting insulin to treat STZ-induced DN in a rodent model. The results showed that although there was no significant synergistic effect between nanocurcumin and insulin, the efficacy of the combination of nanocurcumin and insulin was superior to that of curcumin and insulin alone. SeNPs have been confirmed by many reports to have hypoglycemic effects on rodent models of diabetes. Scholars are not satisfied with only exploring the efficacy of using SeNPs alone, and more studies are focused on the combined application of SeNPs. Metformin is also a first-line drug for the treatment of diabetes. One study proved that the combination of metformin and chitosan SeNPs for 8 weeks was more effective than metformin or chitosan SeNPs alone, which decreased the expression levels of the proinflammatory cytokines TNF-α, IL-6 and IL-1β and restored antioxidant capacity [94]. Sildenafil is widely used to treat angina pectoris and erectile dysfunction and is also used for the experimental treatment of cancer and oxidative stress in mice. In one study, SeNPs in combination with sildenafil were evaluated in the treatment of DN in rats, which reduced oxidative stress and inhibited the expression levels of HMGB1, TNF-α, MCP1, NF-κB and IL1β [95]. Bee venom (BV) has also been reported to have hypoglycemic effects. BV combined with SeNPs can reduce blood glucose, serum BUN, creatinine and C-reactive protein in STZ-induced DN rats and increase serum albumin concentrations [96]. In addition, the combination of rutin and SeNPs can upregulate Nrf-2/HO-1 and downregulate the JAK-2/STAT3 pathway, thereby controlling oxidative damage, inhibiting the increase in IL-6 and TNF-α, and playing a role in renal protection in STZ-induced DN [97]. In short, the combined application of nanotechnology and drug therapy is superior to single therapy, which can not only reduce the blood sugar of a rodent model of DN more effectively but also delay the progression of DN.

#### Therapeutic effect of nanodrug carriers on DN

Cerium, as a rare earth element, has a unique antioxidant effect and can scavenge excessive free radicals under physiological conditions, thereby inhibiting oxidative stress. Cerium oxide, also known as ceria particles, has the ability to clear reactive oxygen species (ROS). Hollow mesoporous nanocomposite particles perfectly retain the antioxidant capacity of ceria particles and have a high drug loading rate because of their surface area, pore volume, large porosity and stability in solvents. This nanocomposite with an oxidative stress targeting function loaded with metformin has hypoglycemic and antioxidant effects, which can prevent the pathogenesis of ROS-related DN [98]. Angiotensin II receptor antagonists have been proven to delay the progression of renal disease in diabetes patients, possibly because hyperglycemia increases angiotensin II production and oxidative stress, which can lead to diabetic complications, including DN [99]. In one study, the therapeutic potential of eprosartan mesylate-loaded nanobilosomes for treating DN was evaluated. This nanocarrier can reduce oxidative stress and alleviate AT1R, inducible NO synthase (NOS), and TGF-β expression to achieve the goal of renal protection in DN [100]. In addition, adropin encapsulated in ROS-responsive nanocapsules may improve renal injury in DN by controlling blood glucose and lipid levels, inhibiting excessive production of ROS and protecting mitochondria [101].

The main function of podocytes as glomerular cells is glomerular filtration. Proteinuria caused by podocyte injury is an important feature of DN. Many studies have focused on targeted drug delivery to podocytes [87, 102, 103]. Some studies have shown that NPs loaded with DXM can be used for the treatment of primary GN [46, 47, 52]. Similarly, DXM-loaded NPs have been proven to be able to repair podocytes and thus can be used in DN treatment [104]. Chitosan has a hydrophilic adhesive structure and can be loaded with hydrophobic drugs. Loaded all-trans retinoic acid (ATRA) chitosan/triphosphoric acid lipid hybrid NPs have a higher oral absorption rate and better therapeutic effect than ATRA [105]. MiRNA-30a is responsible for podocyte homeostasis. Under DN conditions, miRNA-30a inhibition by the hyperglycemia-induced Notch signaling pathway can lead to podocyte injury and apoptosis. Nanocomplexes target exogenous miRNA-30a to podocytes, which can improve albuminuria and podocyte damage [104]. In DN, dyslipidemia may lead to lipoprotein imbalance and mesangial hyperexpansion. He et al. [106] developed a dual targeted treatment for abnormal cholesterol metabolism and the mesangial inflammatory response. Encapsulating liver X receptor agonists in synthetic high-density lipoprotein nanodiscs can effectively remove excess lipids in mesangial cells, improve inflammation, and restore normal kidney function in DN treatment. In summary, nanocarriers can accurately transport drugs and improve bioavailability so that drugs can have a better therapeutic effect.

#### Therapeutic effect of NPs loaded with natural compounds on DN

Natural compounds extracted from plants are widely used to treat various diseases. The active ingredients extracted from plants have better safety and fewer side effects [107].

Rhein, the main active ingredient of rhubarb, is an anthraquinone derivative. Rhein has superior characteristics, such as anti-inflammatory, antibacterial, anticancer, antiviral, and antioxidant properties, but its poor water solubility limits its clinical applicability [108]. Nanocarriers loaded with rhein can solve the problems of low bioavailability, reduced distribution in the kidneys, and adverse reactions. NPs with polyethylene glycol (PEL)-polycaprolactone (PCL) as the core can not only be targeted and transported to the kidneys but also exhibit good renal distribution. Rhein-loaded poly-γ-glutamic acid-coated polymeric NPs [109] and kidney-targeted rhein-loaded NPs with sizes in the range of 30–80 nm for DN can be distributed to the kidneys and enhance renal cellular uptake through the glomerular filtration membrane [110]. Similarly, polyethylenimine (PEI) is a widely used carrier that has proton aminable groups and, to some extent, shows the kidney distribution of gene transmission [111]. Polymer NPs synthesized by PEI and PEL-PCL and loaded with rhein can be effectively absorbed by cells and have therapeutic effects [112]. In addition, stable ultrasmall colloidal nanomaterials with PEL (5–30 nm) can penetrate the GFB and release DXM [113]. In another study, the expression of apical sodium-dependent bile acid transporters mediated the absorption of nutrients, and polymers modified with deoxycholic acid exhibited lower cytotoxicity and higher permeability. The synthesized deoxycholic acid-conjugated NPs have relatively high embedding efficiency (90.7 ± 0.73) % and drug loading efficiency (6.5 ± 0.29) %. In vivo experiments demonstrate a significant improvement in oral bioavailability [114].

Nanoliposomes are colloidal structures composed of double-layer membranes that can be used to encapsulate drugs that are not easily soluble in water. Calycosin is widely used to relieve hypertension, inflammation, diabetes and cancer. The calycosin-loaded nanoliposomes have a stable structure and fully utilize their abilities to restore mitochondrial function [115]. The studies of Zhu et al. [116] and Yang et al. [117] have both demonstrated that the liposome delivery system of anti-inflammatory plant extracts (sinomenine/silymarin) can regulate the JAK2/STAT3/SOCS1 and TGF-β/Smad signaling pathways and inhibit inflammation-related proteins, thereby improving renal injury. Similarly, ergosterol-loaded nanostructured lipid carriers have a relative oral bioavailability that is 277.56% higher than that of ergosterol, which more effectively inhibits high glucose-stimulated mesangial cell proliferation and extracellular matrix accumulation [118]. In brief, natural compounds can act on different targets of inflammation and pathological damage. Compared with pure natural compounds, natural compound nanodrugs have the advantages of targeting to the kidney and good kidney distribution, which significantly improves the ability to fight DN.

### The role of nanomaterials in the treatment of LN

**Fig. 5:**
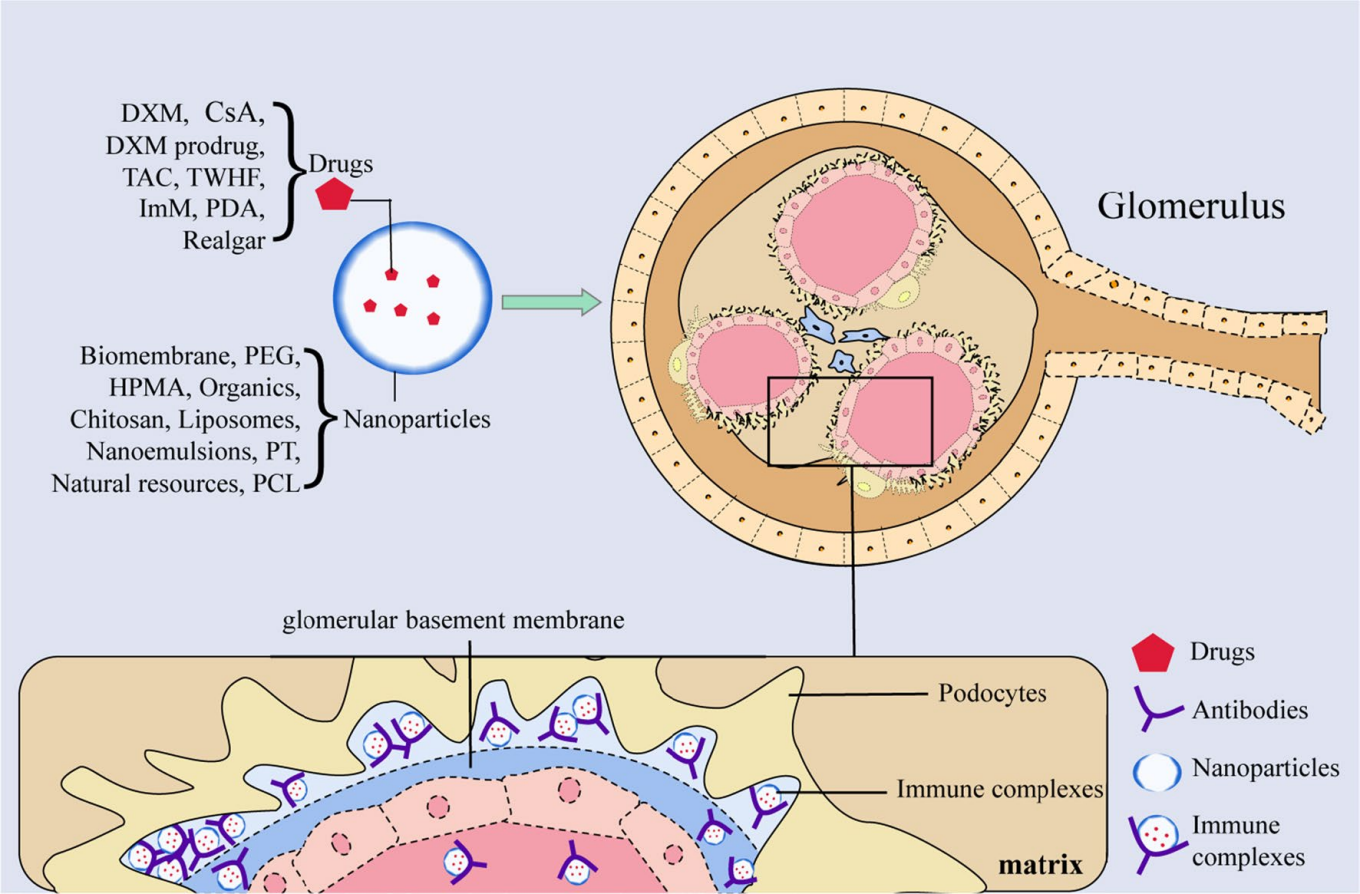
Treatment of nanomaterials in LN. In the treatment of LN, a nanodrug delivery system can target the kidney and form an immune complex. *LN*  lupus nephritis; *CsA*  cyclosporine A, *TAC*  tacrolimus, *PDA*  polydopamine, *PEG*  polyethylene glycol, *HPMA*  P-Dex9 (N-(2-hydroxypropyl) methacrylamide, *TWHF*  tripterygium wilfordii, *ImM*  imatinib mesylate, *PCL*  polyethylene glycol cationic liposome

Systemic lupus erythematosus (SLE) is an autoimmune disease that involves multiple systems. There is an enormous difference between the clinical signs of the disease and the performance of hematology examination, and there is no standardized method to define the response to treatment [119]. LN is one of the most serious complications of SLE. The treatment of LN usually uses hormonal and immunosuppressive therapy, but the effect is not satisfactory and remains an important cause of death in SLE patients [120]. Nanodrug delivery systems can target various drugs to the kidneys (Table 4) (Fig. 5).

**Table 4:** Application and efficacy of nanomaterials in lupus nephritis

Material	Particles	Loaded substance	Therapeutic Effects	Ref
Biomembrane	IFN-embrane Effectsicacy of nanomaterials in efficacyectid profiles in renal tissuesati	DXM	Improve current SLE treatment efficacy, minimizie systemic side effects	[124]
PEG	Polyethylene glycol-based macromolecular prodrug (ZSJ-0228)	DXM prodrug	Local anti-inflammatory/immunosuppressive effects and improve safety	[125]
HPMA	ZSJ-0228 and P-Dex9 (N-(2-hydroxypropyl) methacrylamide copolymer-based dexamethasone prodrug(P-Dex)	DXM prodrug	Reduce proteinuria	[126]
Organics	Biodegradable ligand-conjugated nanoparticles(P71Ns-gambogic acid)	CsA	Increase targeted drug delivery and improve bioavailability	[130]
Chitosan/Micelles	Ethylene Glycol Chitosan Nanomicelles Loaded with Tacrolimus	TAC	Reduce renal inflammationrenal dysfunction, proteinuria and histological injury	[132]
Liposomes	Nanostructured lipid carrier loaded with Tripterygium wilfordii	TWHF	Reduce the collagen content of the renal interstitial cells and remove MCP-1 deposited	[133]
Nanoemulsions	Nanoparticle containing ImM	ImM	Reduce potential for toxicities, enhance drugs accumulation in kidney	[135]
PT	Npolydopamine (PDA)-based nanocarrier modified with Fe_3_O_4_ and Pt nanoparticles (PDA@Pt-Fe_3_O_4_)	PDA	Reduce inflammation, potential for photoacoustic/magnetic resonance dual-mode imaging	[138]
Natural resources	Nanoparticles loaded with Realgar	Realgar	Reduce anti-dsDNA, IgG, IgM, BUN, Cr, IFN-inand proteinuria	[139]
DNA	INH-ODN-DNA nanoflower	INH-ODN DNA	Decrease autoantibodies, reduce cytokine secretion	[140]
PCL	Polyethylene glycol cationic liposome related pDNA and siRNA	pDNA and siRNA	Nucleic acids may exacerbate the symptoms in SLE patients who have preexisting anti-nuclear antibodies	[141]

*CsA* cyclosporine A, *TAC* Tacrolimus, *PDA* Polydopamine, *PEG* polyethylene glycol, *ImM* Imatinib mesylate, *HPMA* P-Dex9 (N-(2-hydroxypropyl) methacrylamide, *TWHF* Tripterygium wilfordii, *PCL* polyethylene glycol cationic liposome

#### The therapeutic effect of nanocarriers loaded with DXM and DXM prodrugs on LN

Prodrugs are inactive compounds in vitro that undergo catalysis or activation in the body before exerting pharmacological effects. They have the abilities to improve pharmacokinetic properties, improve solubility, reduce drug toxicity, and increase the specificity of the site of action [121, 122]. Glucocorticoids are widely used in the clinical management of LN. However, the side effects of long-term use seriously affect its efficacy. Recent studies have shown that prodrug administration in the form of NPs has gradually become a research direction worth exploring [123]. In a study, cancer cell membrane-coated NPs were used to alleviate autoimmune diseases by harnessing the immunosuppressive effects of tumor cells. Researchers prepared IFN-γ-treated MHC class I-deficient cancer membrane-coated NPs containing DXM, a functionally driven, disease-related CD4+ T-cell-targeted drug delivery platform, which could reduce urine protein and serum creatinine levels in LN mice [124]. In another study, a PEL-based macromolecular prodrug (ZSJ-0228) of DXM with the characteristic of self-assembly into micelles in water was applied to female NZB/WF1 mice prone to LN. The results showed that it not only has sustained therapeutic effects but also has no significant adverse reactions [125]. In addition, ZSJ-0228 and the N-(2-hydroxypropyl) methacrylamide copolymer-based DXM prodrug (P-Dex) can both improve LN symptoms in lupus-susceptible NZB/WF1 mice and reduce glucocorticoid side effects. In the MRL/LPR mouse model of LN, P-Dex or ZSJ-0228 was more effective in controlling proteinuria and prolonging survival time than the equivalent dose of Dex. However, adrenal atrophy was observed in P-Dex-treated mice but not in ZSJ-0228-treated mice [126]. In short, prodrug administration in the form of NPs can reduce the side effects of glucocorticoids while ensuring the therapeutic effect and even has a better curative effect. It will be a promising method to treat LN.

#### Therapeutic effect of nanocarriers loaded with immunosuppressive drugs on LNs

Ciclosporin A (CsA) is a fungal-derived cyclic peptide and a common immunosuppressive drug. CsA can selectively and reversibly inhibit the immune response mediated by T cells by inhibiting the phosphatase activity of calcineurin [127] and weaken the Ca^2+^-dependent response of leukocytes to proinflammatory stimuli [128]. However, long-term use of CsA treatment may have some side effects, such as newly developed hyperlipidemia, hypertension, and elevated blood creatinine [129]. In one study, a drug delivery system targeting the lymphatic system was designed, and biodegradable ligand-conjugated NPs (P2Ns-gambogic acid (GA)) were synthesized. P2Ns-GA-encapsulated CsAn increased lymphatic drug delivery by 4 to 18 times compared with ligand-free preparations and commercial CsA capsules, respectively. Moreover, the dose of CsA administered was significantly lower than the nephrotoxicity threshold, and only a few treatment-induced renal abnormalities were observed. P2Ns-GA have good transport performance across the gastrointestinal barrier, which may improve transport into the intestinal capillary network or intestinal-related lymphatic tissue, thereby enhancing delivery to the systemic lymphatic circulation and enhancing the therapeutic effect [130]. Other calcium phosphate phosphatase inhibitors, such as tacrolimus, have also been proven to alleviate nephrotoxicity [131]. Weekly use of hydrophobically modified glycol chitosan loaded with tacrolimus nanomicelles can alleviate renal inflammation, proteinuria and tissue damage in LN-positive mice by regulating the TGF-β1/MAPK/NF-κB pathway. Compared to traditional tacrolimus administration, it has the advantages of smaller doses and longer dosing intervals to exert renal protective effects [132]. In addition, the nanostructured lipid carrier loaded with TW extract can effectively reduce the collagen content of renal interstitial cells and remove MCP-1 deposited in the kidney, thus having a therapeutic effect on immune LN mice [133]. In summary, the dosage of nanodrug therapy is smaller than that of traditional drug therapy, which can reduce renal toxicity.

#### The therapeutic effect of nanocarriers loaded with other substances on LN

Imatinib, a common protein kinase inhibitor, has been developed and approved for the treatment of malignant tumors. Some studies have shown that imatinib also has immunosuppressive and anti-inflammatory effects and can be applied to autoimmune diseases, Alzheimer’s disease, Parkinson's disease and other diseases caused by protein kinase activation mutations [134]. A recent study evaluated the renal deposition status of NPs containing imatinib mesylate in an LN mouse model. The pharmacokinetics of nanoformulations showed changes in pharmacokinetic parameters, indicating a decrease in toxicity potential to imatinib mesylate. Compared to naked drugs, MRL/MpJ-Faslrp mice receiving nanoformulations had a threefold increase in renal deposition of imatinib mesylate after 4 h [135]. Polydopamine (PDA) is rich in phenolic groups, which can eliminate ROS produced in inflammatory reactions [136] and has a strong absorption ability in the near-infrared region. Therefore, it can be used as a photoacoustic imaging contrast agent [137]. In a study, a PDA-based nanocarrier modified with Fe_3_O_4_ and Pt NPs was developed and loaded with necrostatin-1, which has dual imaging and therapeutic effects. Necrostatin-1, as an inhibitor of receptor interacting protein 1 kinase, inhibits receptor interacting protein 1 kinase activity and plays an anti-inflammatory role. Pt NPs can catalyze H_2_O_2_ to produce oxygen, thereby counteracting the hypoxic microenvironment of LNs. PDA and Fe_3_O_4_ act as photographic developers for photoacoustic or magnetic resonance imaging (MRI) [138]. In addition, realgar is widely used as a traditional Chinese medicine for various types of inflammation. Xu et al. [139] prepared NPs loaded with realgar to study the impact on LN. Compared with MRL/lpr control mice, mice treated with realgar NPs showed a significant decrease in the serum levels of anti-dsDNA, IgG, IgM, BUN, creatinine, proteinuria, and the inflammatory cytokine IFN-γ.

Natural DNA is compressed into many spatial folds with long skeletons, and DNA nanopolymers with repetitive target sequences are called nanoflowers. Inhibitive oligodeoxynucleotides have immunomodulatory effects and are dual antagonists of TLR7 and TLR9. Among them, IRS661 and IRS869 nanoflowers can lower the autoantibody levels of mice, reduce the secretion of cytokines, and alleviate LN [140]. In addition, intravenous injection of PEL cationic liposome-related pDNA or siRNA into SLE-susceptible mice forms immune complexes with the previous antinuclear antibody, leading to the occurrence of LN [141]. In summary, nanocarriers not only have excellent targeting properties but also play a role in imaging and auxiliary diagnosis. The application of nanodrug delivery systems can increase the therapeutic efficiency of LN, which has promising application prospects.

## Potential treatment strategies for GN

### Nanodrug delivery system of gas signaling molecules

Gas signaling molecules (GSM) are signaling molecules that regulate physiological and pathological mechanisms, including NO, CO, H_2_, and H_2_S [142]. For a long time, people have defined these gases as harmful substances due to their adverse effects on the human body. However, with the advancement of research, researchers have found that the excellent signal transduction function of GSM can be widely applied in the human body, which has changed people’s perception of GSM [143]. NO and its endogenous producer NOS have been proposed as targets of colorectal cancer carcinogenesis regulation, and inducible NOS inhibitors have application value in the targeted therapy of colorectal cancer [144]. H_2_S has a role in neuroprotection and vascular smooth muscle relaxation [145]. Carbon monoxide-releasing molecules (CORM), as a prodrug producing carbon monoxide, can release CO to play an anti-inflammatory and cytoprotective role [146]. GSM has the advantage of reducing systemic side effects through local delivery, resulting in the development of stimulus-responsive nanocarriers [143].

A certain concentration of H_2_ can trigger death receptors on the cell surface, inhibit survival signaling, and downregulate antiapoptotic proteins. Second, H_2_ can selectively eliminate the most cytotoxic ROS by regulating ROS. In addition, H_2_ can inhibit ILs and TNFs, thereby regulating inflammation [147, 148]. The byproducts of H_2_ have almost no side effects and have high safety in clinical applications. In addition, the ultrasmall size of H_2_ is very suitable for nanomaterial drug delivery systems. Some studies have shown that only excessive amounts of materials can produce therapeutic amounts of H_2_. This may have limitations on in vivo application and targeted release, but larger nanoscale materials can be used to eliminate it and increase accumulation at the targeted location [149].

The three gases H_2_S, NO, and CO have been proven to have antioxidant, anti-inflammatory, anti-apoptotic, and anti-proliferative properties [150]. H_2_S produced by cystathionine β-synthase was proven to have anti-inflammatory activity and energy formation [151]. Some studies have shown that H_2_S has potential inhibitory effects on inflammation and oxidative stress [152, 153]. The increase in H_2_S levels in the kidneys can protect the kidneys after ischemia‒reperfusion injury through anti-inflammatory and antioxidant stress [152, 154]. NO and H_2_S have many similarities, and H_2_S can increase endothelial NOS activation and promote phosphorylation through intracellular Ca^2+^ mobilization [155]. Many studies have shown that endogenous and exogenous NO have antifibrotic mechanisms [156, 157]. Adding an NO donor to cultured rat mesangial cells can inhibit the expression of fibrotic genes at the transcriptional level [158]. This has therapeutic potential for scars produced in the end stage of glomerular inflammation.

In comparison to H_2_S and NO, CO is more stable [159]. In one study, researchers used styrene maleic acid copolymer (SMA) to develop a CO nanodrug delivery system. It forms micelles through self-assembly, which can slow and continuously release CO in the cycle. Mice exposed to CsA for 4 weeks experienced severe kidney damage and decreased renal function. The CO nanodrug delivery system inhibits TGF through the NLRP3 inflammasome-dependent β/Smad signaling pathway and significantly improves inflammatory damage and fibrosis [160]. Similarly, MnO_2_ NPs loaded with Fla encapsulated in neutrophil membranes can target inflammation sites and release CO to produce anti-inflammatory effects [161]. The above results indicate that nanodelivery systems have good potential for delivering GSM in the treatment of GN. GSM has become a promising molecular target for the treatment of diseases because of its extensive biological effects. In kidney diseases, gasotransmitters can regulate inflammation and improve inflammatory damage and may have the ability to inhibit fibrosis.

### Application of subminiature nanomaterials in GN

For NPs, one of the key parameters is size, and different sizes of NPs will have different distributions in the body [162, 163]. If only AuNPs smaller than 10 nm can enter the nucleus, AuNPs larger than 10 nm will remain outside the cell [164]. Ultrasmall AuNPs do not induce ROS toxicity and have higher sensitivity and the ability to be excreted quickly through the kidneys. Therefore, ultrasmall AuNPs can be used in MRI, photoacoustic imaging, positron emission tomography, and X-ray scattering imaging for imaging diagnosis [165]. In addition, the coupling of ultrasmall AuNPs with other drugs can become a treatment platform with dual capabilities, including drugs and targeting agents, which have higher therapeutic effects than free drugs [165]. Inflammation and oxidative stress have a close and complex relationship, and many drugs with the potential to treat acute and chronic inflammation have antioxidant stress effects [166]. ROS in inflammatory reactions may promote local damage, delay healing time, and lead to chronic inflammation [167, 168]. In one study, the ability of ultrasmall Cu_5.4_O NPs to clear ROS and alleviate inflammation was evaluated. The results showed that ultrasmall Cu_5.4_O NPs had the characteristics of catalase, superoxide dismutase and glutathione peroxidase simulants. A very small dose of ultrasmall Cu_5.4_O NPs can clear ROS, effectively improving acute injury and promoting wound healing [169]. Our previous studies confirmed that the microminiature zirconium carbide nanodots used in glioma therapy have excellent anti-inflammatory, ROS clearance and renal clearance abilities [170, 171]. Cerium oxide NPs (CeONPs) have strong antioxidant properties, but formulations containing CeONPs cannot be effectively removed from the body. Ultrasmall CeONPs retain their antioxidant properties, effectively clearing ROS, inhibiting macrophage activation, and minimizing their recruitment and infiltration into inflammatory sites, thereby alleviating acute inflammation. Ultrasmall CeONPs are effectively excreted from the body within 24 h after systemic administration, greatly alleviating toxic side effects [172]. In addition, ultrasmall solid lipid NPs with Dex (SAN-Dex) reduced TNF-α, IL-774 and IL-1 levels in lipopolysaccharide-stimulated J6A12 cells. Through oral administration, SAN-Dex still retains its anti-inflammatory activity, but ordinary solid lipid NPs with Dex lose their anti-inflammatory capacity [173]. Based on the ultrasmall size of nanomaterials, they can effectively utilize their original characteristics, such as the ability to clear ROS, antioxidant stress and anti-inflammatory properties, and have advantages such as low toxicity, easy excretion and targeted localization. It is hoped that more effective drugs or materials can be targeted to the glomeruli for more precise treatment of GN.

## Conclusions and future directions

The pathogenesis of GN is complex and has not been thoroughly studied. The treatment of GN is mainly symptomatic treatment, which can rarely cure the disease from the root, mainly slowing down the disease progress and maintaining the status quo. Immunotherapy is used for specific lesions and is accompanied by severe side effects, and steroid resistance and recurrence are also common. When GN progresses to end-stage renal failure, renal replacement therapy is needed. Dialysis treatment needs to be carried out for a long time, which causes a great economic burden to some patients' families. Renal transplant patients are also prone to recurrent GN, leading to transplant failure.

Fortunately, with the widespread development and application of nanodrug delivery systems, targeting NPs to deliver drugs to the kidneys is an emerging and highly promising treatment method. The use of nanomaterial-loaded drugs can accurately target kidney cells, reduce adverse drug reactions, and produce better therapeutic effects. NPs for diagnosis and treatment are constantly being developed, including NPs that integrate diagnosis and treatment at the same time for the purpose of diagnosis, localization and treatment. At present, most of the NPs used for GN are nanoloading platforms, and there are also a few studies on the combination of NPs and first-line drugs for diseases. At the same time, some nanomaterials (mostly metal NPs) have been validated in GN for their inherent anti-inflammatory or antioxidant stress properties.

**Fig. 6:**
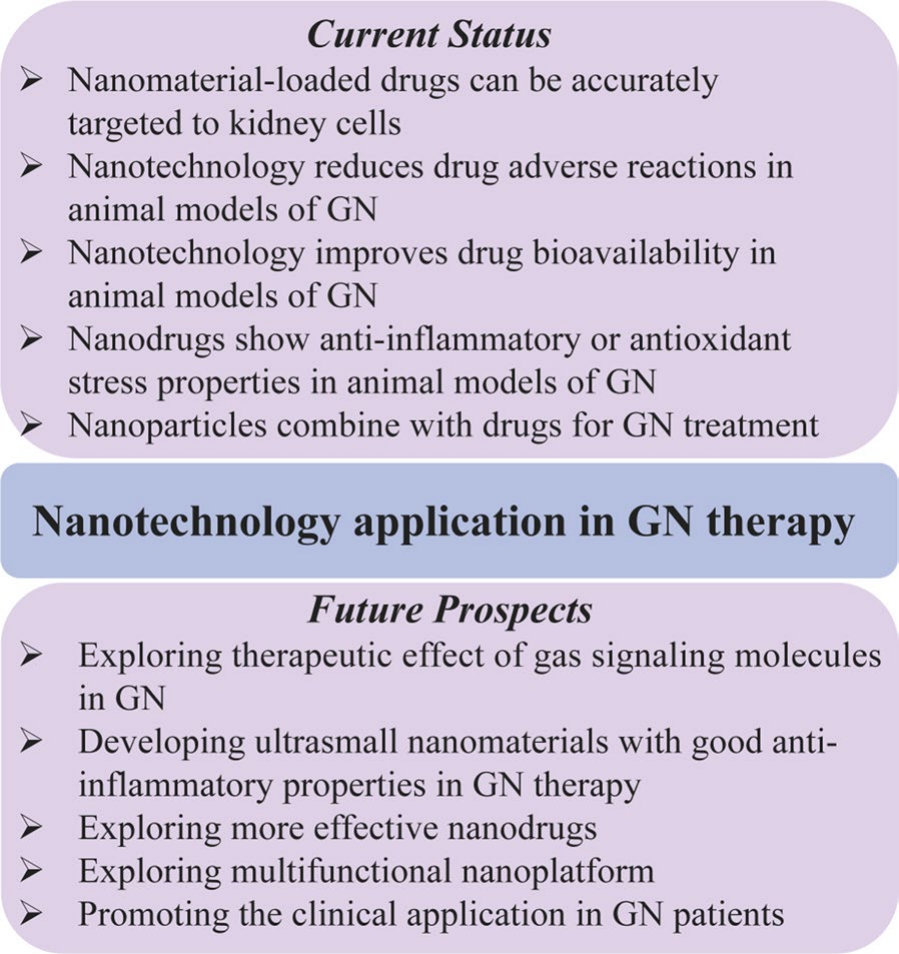
Current status and future prospects of nanotechnology in GN therapy. *GN* glomerulonephritis

Except for DN, there are relatively few high-quality studies and experiments on other types of GN, especially primary GN. Meanwhile, any single treatment method has limitations, and combination therapy and multifunctional nanoplatforms can compensate for the shortcomings of a single therapy and achieve better results. In addition, for example, GSM and ultrasmall nanomaterials have the characteristics of wider distribution and faster metabolism due to their small size. We believe that it is possible to explore GSM nanodrug delivery systems and ultrasmall nanomaterials in GN therapy. Current status and future prospects on nanotechnology application in GN therapy were summarized in Fig. 6.

Based on the contents discussed above, there is reason to believe that future research on GN therapy should be multifaceted and that more attention should be given to the treatment of primary GN, with more attempts to combine NPs with other treatment methods and develop multifunctional nanoplatforms.

## Data Availability

All the data reported in this manuscript is available within the text.

## Abbreviations

GN: Glomerulonephritis
FSG: Focal segmental glomerulosclerosis
IgAN: Immunoglobulin A nephropathy
DN: Diabetic nephropathy
LN: Lupus nephritis
ANCA: Antineutrophil cytoplasmic antibody
anti-GBM: Anti-glomerular basement membrane antibody
ESRD: End-stage renal disease
NPs: Nanoparticles
GFB: Glomerular filtration barrier
GBM: Glomerular basement membrane
GFR: Glomerular filtration rate
ACEis: Angiotensin-converting enzyme inhibitors
ARBs: Angiotensin II receptor blockers
TW: Tripterygium wilfordii
HAS: Human serum albumin
PFs: Peptide fragments
CLT: Celastrol
TP: Triptolide
PF-A_299-585_: PF with amino acid sequence A_299-585_
DXM: Dexamethasone
HA: Hyaluronic acid
MM: Macrophage membranes
BUM: Blood urea nitrogen
CAP: Captopril
HGC: Hydrophobically modified glycol chitosan
miRNAs: MicroRNAs
siRNAs: Short interfering RNAs
EGCG: Epigallocatechin-3-gallate
EPO: Erythropoietin
SeNPs: Selenium nanoparticles
STZ: Streptozotocin
ZnONPs: Zinc oxide nanoparticles
AuNPs: Aurum nanoparticles
AgNPs: Argentum nanoparticles
BV: Bee venom
ROS: Reactive oxygen species
NOS: Inducible nitric oxide synthase
ATRA: All trans retinoic acid
PCL-PCL: Polyethylene glycol-polycaprolactone
PEL: Polyethylenimine
SLE: Systemic lupus erythematosus
CsA: Ciclosporin A
PDA: Polydopamine
Fe_3_O_4_: Ferrosoferric oxide
H_2_O_2_: Hydrogen peroxide
MRI: Magnetic resonance imaging
GSM: Gas signaling molecules
NO: Nitrogen monoxide
CO: Carbon monoxide
H_2_: Hydrogen
H_2_S: Hydrogen disulfide
SMA: Styrene maleic acid copolymer
MnO_2_ NPs: Dioxide manganese nanoparticles
CeONPs: Cerium oxide: nanoparticles
